# What could be the function of the spinal muscular atrophy-causing protein SMN in macrophages?

**DOI:** 10.3389/fimmu.2024.1375428

**Published:** 2024-05-28

**Authors:** Ines Tapken, Nora T. Detering, Peter Claus

**Affiliations:** ^1^ SMATHERIA gGmbH – Non-Profit Biomedical Research Institute, Hannover, Germany; ^2^ Center for Systems Neuroscience (ZSN), Hannover, Germany

**Keywords:** macrophage, monocyte, spinal muscular atrophy, SMA, SMN, SMN gene, neurodegeneration

## Abstract

Spinal Muscular Atrophy (SMA), a neurodegenerative disorder, extends its impact beyond the nervous system. The central protein implicated in SMA, Survival Motor Neuron (SMN) protein, is ubiquitously expressed and functions in fundamental processes such as alternative splicing, translation, cytoskeletal dynamics and signaling. These processes are relevant for all cellular systems, including cells of the immune system such as macrophages. Macrophages are capable of modulating their splicing, cytoskeleton and expression profile in order to fulfil their role in tissue homeostasis and defense. However, less is known about impairment or dysfunction of macrophages lacking SMN and the subsequent impact on the immune system of SMA patients. We aimed to review the potential overlaps between SMN functions and macrophage mechanisms highlighting the need for future research, as well as the current state of research addressing the role of macrophages in SMA.

## Introduction

The Survival Motor Neuron (SMN) protein has been extensively studied in the neuronal system as it causes the developmental and neurodegenerative disease Spinal Muscular Atrophy (SMA). SMN is expressed ubiquitously and SMA is regarded as a multi-system disease. However, the immune system has not been sufficiently analyzed in SMA yet. Macrophages are innate immune cells. Their function is regulated by e.g., alternative splicing, cytoskeletal modulation and signaling. Those mechanisms depend on SMN. Therefore, the role of SMN functions in macrophages and the potential impact on the immune system in SMA patients are yet to be investigated. This review aims to stimulate future research about SMN functions in the immune system focusing on macrophages and their role in SMA pathology.

## Macrophages and monocyte-derived macrophages

Macrophages are innate immune cells forming a network in tissues in which they provide homeostasis by phagocytosis of dying cells, cell debris, bacteria, and immune complexes (1). They can be classified into tissue-resident and blood-derived cells, the latter being mediated by chemokines. Resident macrophages induce tissue-specific metabolic processes, provide a first layer of defense for the acute phase reaction, and activate the early innate immune response. Following this dual characteristic in homeostasis and defense, macrophages become specialized for specific tissues after differentiation and additionally need to change their phenotype and signaling profile rapidly (2). Macrophages start to differentiate during organogenesis either from embryonic yolk sac, fetal liver or from bone marrow. In adults, yolk sac-derived macrophages persist in organs as self-maintaining populations or as bone marrow-derived monocytes in the blood. Monocytes are recruited into an organ upon infection or metabolic defect, where they differentiate into macrophages, respectively, contributing to clearance, wound repair, fibrosis, and angiogenesis (2–4). The classification into M1 and M2 macrophages represents an approximate categorization, which has been divided into subclassifications depending on external stimuli and expression profiles. M1 macrophages are activated by e.g., lipopolysaccharides (LPS) from bacteria, interferon gamma (IFNy) produced by T_H_1 lymphocytes upon inflammation or granulocyte macrophage colony-stimulating factor (GM-CSF). M1 cells display a phenotype with distinct cytokine production, enhanced inflammatory, antimicrobial, and antigen-presenting properties other than monocytes. M2 macrophages are alternatively activated by T_H_2 lymphocytes by interleukin 4 (IL-4) and IL-13 and perform anti-inflammatory activities (5). The different types of macrophages have distinct expression profiles to maintain functional plasticity (6).

## Spinal muscular atrophy and the survival motor neuron protein

The Survival Motor Neuron (SMN) protein is a ubiquitously expressed multifunctional protein. Its central role was recognized by identification of SMN as the disease-causing protein of the neurodegenerative and neurodevelopmental disorder Spinal Muscular Atrophy (SMA), the leading genetic disease of newborn and infants (7). Total loss of SMN is embryonically lethal (8). SMN is expressed from two genes, *SMN1* and *SMN2.* Mutations or deletions in *SMN1* lead to SMA (9). *SMN2* differs from *SMN1* in a crucial base transition resulting in a small amount of functional protein expression failing to compensate the loss of *SMN1* (10). Patients with SMA have different residual amounts of full-length SMN due to copy number variations (CNV) of the *SMN2* gene, which inversely correlate with disease severity (11). SMA has been clinically classified into types 0 to IV defined by age of onset and motor milestones. During embryogenesis and development, SMN levels decrease by cell type-specific needs, but SMN remains to be important also in adults (12–14). SMN has several interaction partners and fundamental functions e.g., in assembly of small nuclear ribonucleoprotein particles (snRNPs) or cytoskeletal regulation (15–17).

In SMA, research focused on protein restoration in the central nervous system (CNS) and the periphery, as the role of this protein is highly important in developmental processes and peripheral tissues (14, 18, 19). Currently, three treatments are available for SMA patients, increasing SMN expression in the central nervous system (CNS) or periphery, respectively (20–22). The therapies prolong the survival of patients, improve the phenotype, but do not cure the disease. SMA is now considered as a multi-system disease reflecting its ubiquitous expression pattern (23, 24).

**Figure 1:**
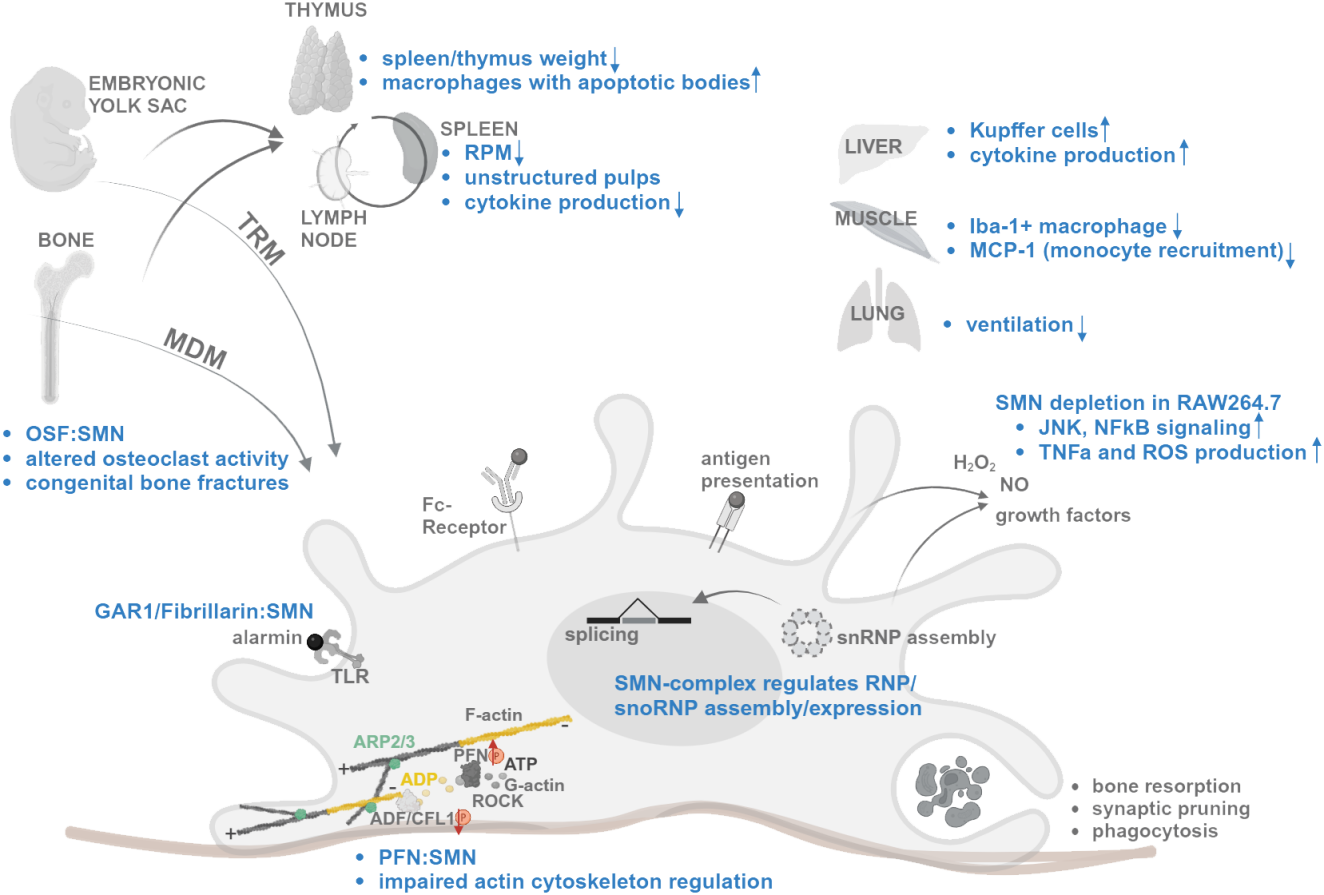
Schematic summary of SMN functions and changes in SMA (blue) affecting mechanisms of macrophage differentiation, migration, splicing regulation, activation, and tissue specific functions. Interaction denoted as “:”. Increased and decreased expression/activity denoted as arrow. For references, see text. Created with BioRender.com.

We hypothesize that functions of SMN affect key mechanisms in monocyte-derived and tissue-resident macrophages. Here, we review the current knowledge about SMN functions to stress its potential role in mechanisms important in macrophages (**Figure 1**). We aim to highlight selected functional aspects of macrophages which may be of relevance for SMN involvement. Moreover, we want to point out the knowledge gap of SMN’s role in macrophages.

## Development

Macrophages emerge early during embryogenesis forming a structural network in developing organs. These tissue-resident macrophages (TRM) are mainly self-renewal. During postnatal tissue maturation, hematopoietic stem cell-derived monocytes (HSC) differentiate into tissue-specific monocyte-derived macrophages (MDM) (25). Several tissues are dependent on regular replenishment during adulthood. In certain tissues, fetal-derived and HSC-derived macrophages coexist in defined anatomical niches contributing to specific function during both homeostasis and inflammation (26). One of the best studied organs with regard to macrophage niches and function is the central nervous system (CNS) (26). In summary, the CNS harbors microglia, a type of macrophages, in the parenchyma and tissue-resident macrophages at ventricles, meninges, and perivascular space (27). The whole adult population of microglia is derived from fetal progenitors and undergoes long-lived self-renewal. They depend on niche factors to maintain their roles in homeostasis, surveillance, and CNS development (28). It has been recently shown that skull bone marrow is a source of MDM progenitors. Those can directly migrate during inflammation from the skull bone marrow via dural channels into the inflamed CNS parenchyma (29). Functional or molecular alteration of MDMs from skull bone marrow in SMA has not been described yet. SMA is not only a neurodegenerative disease but affects all tissues including bone and its development (30, 31). Tissue-resident macrophages in the bone are osteoclasts. SMN interacts by direct protein-protein interaction with osteoclast stimulatory factor (OSF) resulting in altered signaling in osteoclast formation and activity (32, 33). Since SMN is expressed in high amounts during embryonal development, it can be expected that SMN is needed especially during differentiation of cells (12, 34). Whether SMN loss affects macrophage development and bone marrow-derived renewal remains unknown. This may be particularly relevant as congenital bone fractures occur in severe SMA patients (30). SMA patients with type I-III also can show fragility fractures with lower bone mineral density (35).

## RNA metabolism

SMN functions impact several RNA metabolic processes, e.g. spliceosomal snRNP assembly in the cytoplasm mediated by the SMN-complex comprising SMN, GEMIN2–8 and UNRIP (36, 37). SMN localizes to Cajal bodies (CB) and regulates the integrity of nuclear Gemini of Cajal bodies (gems) (38, 39). It also stimulates alternative splicing of pre-mRNA mediated by snRNPs in the nucleus (15). Macrophages comprise different types of membraneless organelles to ensure a robust immune response controlling the kinetics of inflammatory gene expression. The nuclear paraspeckle regulates innate immune gene expression through the nuclear retention of RNA via RNA-protein interaction including Neat1 long non-coding (lnc) RNA (40, 41). Another subnuclear body is the interleukin-6 (IL-6) and -10 (IL-10) splicing activating compartment (InSAC) controlling processing of interleukin RNAs in immune response. IL-6 and IL-10 regulate pro- and anti-inflammatory homeostasis. Thereby, DNA-binding protein-43 (TDP-43) scaffolds interleukin RNAs and recruits spliceosomal components such as snRNPs from Cajal bodies (CBs) under inflammatory conditions (42). Therefore, CBs are disrupted during inflammation. This process includes a switch of binding affinity of SMN’s interaction partners, leading to reduced binding to TDP-43 and stronger binding to Coilin (43).

Transcriptional regulation and alternative splicing control specific gene expression profiles of different types of macrophages (44, 45). Regulation of macrophage differentiation by splicing has been analyzed by RNAseq in primary macrophages and THP-1 cells, a human monocyte cell line. RNA binding proteins (RBPs) change their expression profile during differentiation and activation of monocytes and macrophages. Induction results in regulation of RNA-binding proteins such as SNRPE, GEMIN2, RAVER2 and ELAVL4 (44). GEMIN2 is the main interactor of SMN in the SMN-complex in snRNP assembly (37). Additionally, SMN regulates the assembly of small nucleolar ribonucleoprotein complexes (snoRNPs). Small nucleolar RNA (snoRNA) is a noncoding RNA which is bound to specific proteins to form snoRNPs. Those perform posttranslational modifications of non-coding RNAs and ribosomal RNAs (46). During this process, SMN interacts with snoRNP proteins as ribonucleoprotein complex subunit 1 (GAR1) and Fibrillarin (47). Those are members of the alarmin protein family which activate Toll-like receptors on the cells surface of antigen presenting cells (APC) such as macrophages (48). Activation of macrophages results in regulation of several snoRNAs (49).

## Cytoskeleton and actin

The actomyosin cytoskeleton maintains cell shape, drives cell movement, and controls cellular mechanosensing, signaling and cell-cell communication (50). Key elements are actin filaments, actin binding proteins (ABP) and myosin (51). Assembly and disassembly are important to regulate cellular functions such as membrane protrusion, endocytosis, exocytosis, vesicle trafficking, organelle positioning and phagocytosis (52). Actin filament assembly is regulated on the barbed end of filamentous actin (F-actin) by ATP-bound actin interacting with profilin (53). Branching starts by binding of the Arp2/3 complex bound to monomeric globular (G-) actin on the side of a pre-existing F-actin filament (54). Capping protein determines the growth and filament turnover by hydrolysis of ATP bound to each actin-monomer (55). Actin depolymerizing factor (ADF)/cofilin, which is regulated by Rho-kinases, promotes the phosphate and ADP-actin dissociation from a filament. G-actin recycling is regulated by profilin (56). Profilins have binding sites for G-actin, phosphatidylinositol 4,5-bisphosphate (PIP2) and poly-L-proline (PLP) (57). SMN comprises PLP stretches and binds profilin1 and 2a (58). SMN depletion results in profilin2a hyperphosphorylation leading to dysregulated actin dynamics. Profilin2 regulation by the Rho-associated coiled-coil kinase (ROCK) is activated in SMA leading to an impaired actin cytoskeleton regulation and neurite outgrowth (58–61). Macrophages dynamically alter their actin cytoskeleton to migrate and engulf material. Thereby, the Arp2/3 complex is important for extending large protrusions as lamellipodia (62, 63). However, macrophages are capable to use filopodia for phagocytosis in a less motile state upon Arp2/3-deficiency (64). Therefore, Arp2/3 is crucial for cell migration (65). In macrophage activation, the actomyosin structures around the nucleus first become contractile and later spread out with less association between actin and myosin. This process influences the function of macrophages. The importance of actin regulation for the function of leukocytes, white blood cells including monocytes, has been shown by lower chemokine levels and MHCII surface localization upon Arp2/3 deficiency (66). In actinopathies, such as the Wiskott-Aldrich syndrome, in which mutation in Arp2/3-activating nucleation promoting factor (NPF) WASP results in autoinflammation and immunodeficiency, leukocytes are profoundly affected (66). Interestingly, SMN affects the localization of RPS6 mRNA to the plasma membrane of fibroblasts, where RPS6 mRNA associates with calveolin-1 (67). Calveolin-1 is a key component of membrane dynamics and plays a critical role in the differentiation of monocytes into macrophages (68). Due to the interaction of SMN with profilin and association with calveolin-1 during cytoskeletal modulation, we hypothesize that SMN loss also affects the migratory behavior of macrophages and engulfment of cellular debris.

## Signaling: inflammatory response, tissue recruitment and lymphoid organs

Signal transduction regulates the functional plasticity of macrophages to respond to different conditions and exerts their specific functions. Macrophages are tightly regulated in physiological conditions by signaling pathways, which can react to numerous environmental stimuli and drugs [reviewed in (69)]. This functional plasticity is regulated by c-Jun amino terminal kinase (JNK) and activator of transcription pathways such as Wnt and Notch signaling (69). The inflammatory responses are mediated by nuclear factor-kappaB (NFκB) and JNK. SMN protein depletion in the mouse macrophage-like cell line RAW264.7 activates those pathways and increases TNF-α and reactive oxygen species production (70). JNK and NFκB signaling can be inhibited physiologically by TRAF6, which has been found to be a direct interactor of SMN (71). Therefore, SMN may have a regulatory function in inflammatory and oxidative stress response in macrophages (70). During neurodegeneration, synaptic stripping is a microglia-mediated process enabling removal of damaged pre-synaptic axon ends by controlled phagocytosis to ensure cell repair (72). In SMA, microglia exhibit an enhanced inflammatory profile negatively affecting motoneuron survival (2, 73).

Recruitment of monocytes to inflammatory regions is regulated by chemokine signaling. In symptomatic SMA (Smn^−/−^;SMN2^tg/tg^) mice, Iba-1+ macrophage density was reduced in SMA muscles although apoptosis and subsequent recruitment was stimulated. Before muscle atrophy, newborn mice showed no difference in densities. MCP-1 is a factor involved in monocyte recruitment and was significantly downregulated in muscles of symptomatic mice (74). MCP-1 secretion is decreased in SMN-deficient cultured astrocytes, which fail to support wild type motor neurons. Restoration of MCP-1 results in neurite outgrowth from motor neurons (75). This defect in recruitment has not been observed in spleen and liver of SMA mice. However, the weight of spleen and thymus, two lymphoid organs, was reduced by 4- to 6-fold in SMA compared with controls. Other peripheral tissues such as kidney and liver as well as the whole body were only 2-fold reduced in weight (74). Infiltration of macrophages was present in intestine of severely affected SMA mice (denoted as the Taiwanese mouse model) showing an aberrant number of neurons in the enteric nervous system (ENS). This infiltration was reduced after increase of SMN expression via antisense oligonucleotide (ASO) treatment (20).

Astrocytes and glial cells exert functions in SMA (reviewed by 76). Gliosis, defective signaling between astrocytes and neurons, structural dysfunctions of astrocytes, increased activation of microglia, and infiltration of peripheral immune cells may promote pathogenesis in SMA. SMN restoration in those cells influences SMA pathology suggesting a role of SMN beyond the motor neuron (76). Cell type specific loss of SMN in iPSC-derived microglia results in a `amoeboid morphology`, displaying a changed reactive transcript profile, increased migration and phagocytotic activity (77).

In different SMA mouse models, abnormalities have been found in the primary lymphoid organ thymus and in the secondary lymphoid organs spleen and mucosa-associated lymphoid tissue (MALT) (73, 78, 79). However, there is still limited knowledge about alterations of lymph nodes or bone marrow in SMA. The spleen displayed developmental defects in white pulp formation, showed increased cell death and fibrotic tissue structures with lack of collagen in the severe (Taiwanese) SMA mouse model at mid-symptomatic [postnatal day 5 (P5)] stage (79). Intriguingly, the number of yolk-sac derived red pulp macrophages (RPM) was reduced at symptomatic P12 and at presymptomatic P2 stage in the spleen of the SMNΔ7 SMA mouse model (80). Macrophages localize diffusely in *Smn^2B/-^* spleen at P4 and infiltrate the white pulp with increased unstructured localization at later time points. The mechanisms behind those differences in mouse models are currently unclear but could be a consequence of different kinetics of pathogenesis. In the thymus of P19 intermediate *Smn^2B/^* ^-^ mice, an organ for lymphocyte maturation, more macrophages with apoptotic bodies have been found in mild and severe SMA models. Cytokine expression profiles changed in P19 thymus from *Smn^2B/^* ^-^ mice. Both organs were decreased in size in *Smn^2B/^* ^-^ and were rescued by introduction of one *SMN2* copy (78). Changes in cytokine production also have been found in early and late symptomatic SMNΔ7 mice. These showed a selective reduction of the red pulp (RP) and RPM, whereby the white pulp was preserved. Pro-inflammatory cytokines were produced in early and late symptomatic mice and after LPS challenge, the cytokine production was reduced in astrocytes and splenocytes (not for IL-1ß) (80). Taken together, SMA mouse models present different structural and organizational defects in lymphatic tissues, which may depend on migratory functions of cells but also chemokine production, as these organs are structured by gradients. The high expression of SMN in lymphoid organs of wild type mice compared to tissues strongly associated with SMA (skeletal muscle, spinal cord) underscores the need to evaluate its role in immune cells during development and adulthood (78). SMN is expressed in high amounts during embryogenesis and decreases over time (12, 81). The tissue-specific need of SMN expression may influence immune cells differently, as immune cells, also monocyte-derived macrophages, differentiate throughout life.

## Other peripheral defects in SMA

Macrophages are present in both neuronal and non-neuronal tissues as microglia (CNS), osteoclasts (bone), Kupffer cells (liver), alveolar macrophages (lung), Langerhans cells (skin) and histiocytes (connective tissue) (82). In liver of presymptomatic Taiwanese SMA mice, the number of Kupffer cells was increased reflecting early systemic inflammation with increased expression of proinflammatory cytokines (also in lung and intestine) including Interleukin-1β (IL-1β), IL-6 and TNF-α (83). Those cytokines have also been found to be upregulated in SMA mouse models and other tissues, e.g. in spinal cord of SMA patients (however, with a decrease of IL-6) (78, 84).

The lung is one major organ displaying a connection with the outer environment of the body. Thus, the lung has several immune mechanisms, such as mechanical defense by surfactant, immune sensing by airway epithelial cells, and bronchus-associated lymphoid tissue (BALT). The lung can activate immediate adaptive immune functions (85). Macrophages are the most frequent immune cells present (86). In newborn lungs, fetal liver and yolk sac-derived macrophages are present. Bone marrow-derived macrophages start to immigrate and differentiate into alveolar macrophages (AM), essential for clearance and surfactant-regulation by local secretion of the cytokine GM-CSF (2, 87, 88). Histological inspection of SMA indicates lesions of alveolar septa in the lung that could not be rescued by general increase of SMN levels through histone deacetylase (HDAC) inhibition by JNJ-26481585 (89). This inhibitor also activates NFκB and IL-1β production in RAW 264.7 mouse macrophage cells (90). Lung and pulmonary phenotypes have been described in clinical studies, showing a decline in ventilation capacity with a stabilization in adulthood and positive correlation with therapy (91, 92). Most studies trace defects in lung function to the degeneration of intercostal muscles and diaphragm function, but SMN-dependent primary defects and therapeutic assessment of those have not been studied yet. The involvement of macrophages in lung and other affected tissues in SMA are yet to be elucidated and in focus of future research.

Defects in lymphoid organs and CNS, bone and liver, indicate a putative regulatory role of macrophages being impaired in SMA. It is still unclear whether SMA patients experience alterations of immune responses and a putative change upon treatment. Interestingly, SMA patients treated with the ASO Nusinersen showed macrophages with dark inclusions in the cerebrospinal fluid (CSF). After treatment, the CSF showed unique inclusions comprised of glycosaminoglycanes in macrophages, which were most likely monocyte derived (93, 94). Another study reported an increase in IL1ß, IL23 and IL6, which can be produced by macrophages, in sera of pediatric and adult SMA patients type I-III treated with Nusinersen (95). These findings underline the need for further analyses of the current treatments with regard to their impact on the immune response.

## Conclusion

It is currently unknown whether macrophages play a significant role in SMA, thereby impacting various organ function and immune responses. The intricate balance between tissue-resident and blood-derived macrophages, their diverse roles in tissue homeostasis and defense, and their rapid adaptability underscore their importance for tissue homeostasis. The Survival of Motoneuron protein (SMN) is the disease-determining factor of SMA and regulates critical mechanisms for cellular functions such as translation, splicing and cytoskeletal modulation. The role of SMN in macrophages has been studied only marginally showing a role of SMN in signaling of macrophages and osteoclast development. *Vice versa*, macrophages are involved in the pathogenesis of SMA a multi-organ disease. The discussed high overlap in molecular mechanisms important in macrophages and regulated by SMN emphasize the need for a detailed analysis of the role of SMN in monocytes, macrophages, and cells of the lymphatic system. This addresses the broader systemic aspect of SMA, potentially critical for SMA patients in the future.

## Author contributions

IT: Conceptualization, Funding acquisition, Methodology, Visualization, Writing – original draft, Writing – review & editing. ND: Conceptualization, Funding acquisition, Supervision, Writing – review & editing. PC: Conceptualization, Funding acquisition, Methodology, Project administration, Supervision, Validation, Writing – review & editing.
